# Corrigendum: Motivational, proteostatic and transcriptional deficits precede synapse loss, gliosis and neurodegeneration in the B6.*Htt*^*Q111*/+^ model of Huntington’s disease

**DOI:** 10.1038/srep44960

**Published:** 2017-03-28

**Authors:** Robert M. Bragg, Sydney R. Coffey, Rory M. Weston, Seth A. Ament, Jeffrey P. Cantle, Shawn Minnig, Cory C. Funk, Dominic D. Shuttleworth, Emily L. Woods, Bonnie R. Sullivan, Lindsey Jones, Anne Glickenhaus, John S. Anderson, Michael D. Anderson, Stephen B. Dunnett, Vanessa C. Wheeler, Marcy E. MacDonald, Simon P. Brooks, Nathan D. Price, Jeffrey B. Carroll

Scientific Reports 7: Article number: 4157010.1038/srep41570; published online: 02 08 2017; updated: 03 28 2017

This Article contains a typographical error in the Methods section, under the subheading “Library construction, RNA Sequencing and RNASeq analysis”, where:

“The fastq files were aligned using the default parameters of SNAPR^50^ (https://github.com/PriceLab/snapr) against the GRCh38 genome assembly, along with the transcriptome assembly gtf file from Ensembl, GRCh38.75”.

should read:

“The fastq files were aligned using the default parameters of SNAPR^50^ (https://github.com/PriceLab/snapr) against the GRCm38 genome assembly, along with the transcriptome assembly gtf file from Ensembl, GRCm38”.

