# Supplementary material for: Motivational, proteostatic and transcriptional deficits precede synapse loss, gliosis and neurodegeneration in the B6.HttQ111/+ model of Huntington’s disease
Source: Sci Rep. 2017 Feb 8;7:41570. doi: 10.1038/srep41570 (PMC5296868; doi:10.1038/srep41570)
Supplement: Supplementary Figures [file srep41570-s1.doc]

# **Supplemental Material**

# **Motivational, proteostatic and transcriptional deficits precede synapse loss, gliosis and neurodegeneration in the B6.*Htt^Q111/+^* model of Huntington's disease**

Robert M. Bragg ^1+^, Sydney R. Coffey ^1+^, Rory M. Weston ^1,2^, Seth A. Ament ^3,6^, Jeffrey P. Cantle ^1^, Shawn Minnig ^1^, Cory C. Funk ^3^, Dominic D. Shuttleworth ^1^, Emily L. Woods ^1^, Bonnie R. Sullivan ^1^, Lindsey Jones ^1^, Anne Glickenhaus ^1^, John S. Anderson ^1^, Michael D. Anderson ^1^, Stephen B. Dunnett ^5^, Vanessa C. Wheeler ^4^, Marcy E. MacDonald ^4^, Simon P. Brooks ^5^, Nathan D. Price ^3^, and Jeffrey B. Carroll ^1,4*^

# **Affiliations**

^1^ Behavioral Neuroscience Program, Department of Psychology, Western Washington University, Bellingham, WA, USA

^2^ Department of Pharmacology and Toxicology, Virginia Commonwealth University, Richmond, VA, USA

^3^ Institute for Systems Biology, Seattle, WA, USA

^4^ Center for Human Genetic Research, Massachusetts General Hospital, Harvard Medical School, Boston 02114, Massachusetts

^5^ The Brain Repair Group, Cardiff University School of Biosciences, The Sir Martin Evans Building, Museum Avenue, Cardiff, Wales CF10 3AX, United Kingdom

^6^ Institute for Genome Sciences and Department of Psychiatry, University of Maryland School of Medicine, Baltimore, MD 21201

###
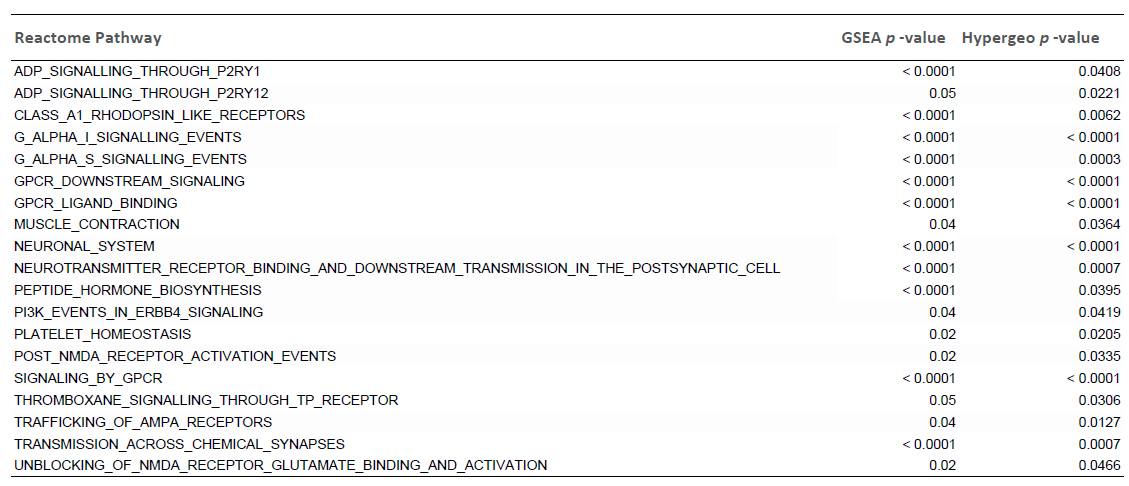
***Table S1.*** *Nineteen common reactome pathways with nominal enrichment p-values below 0.05 in both Hypergeometric and GSEA analysis.*

#
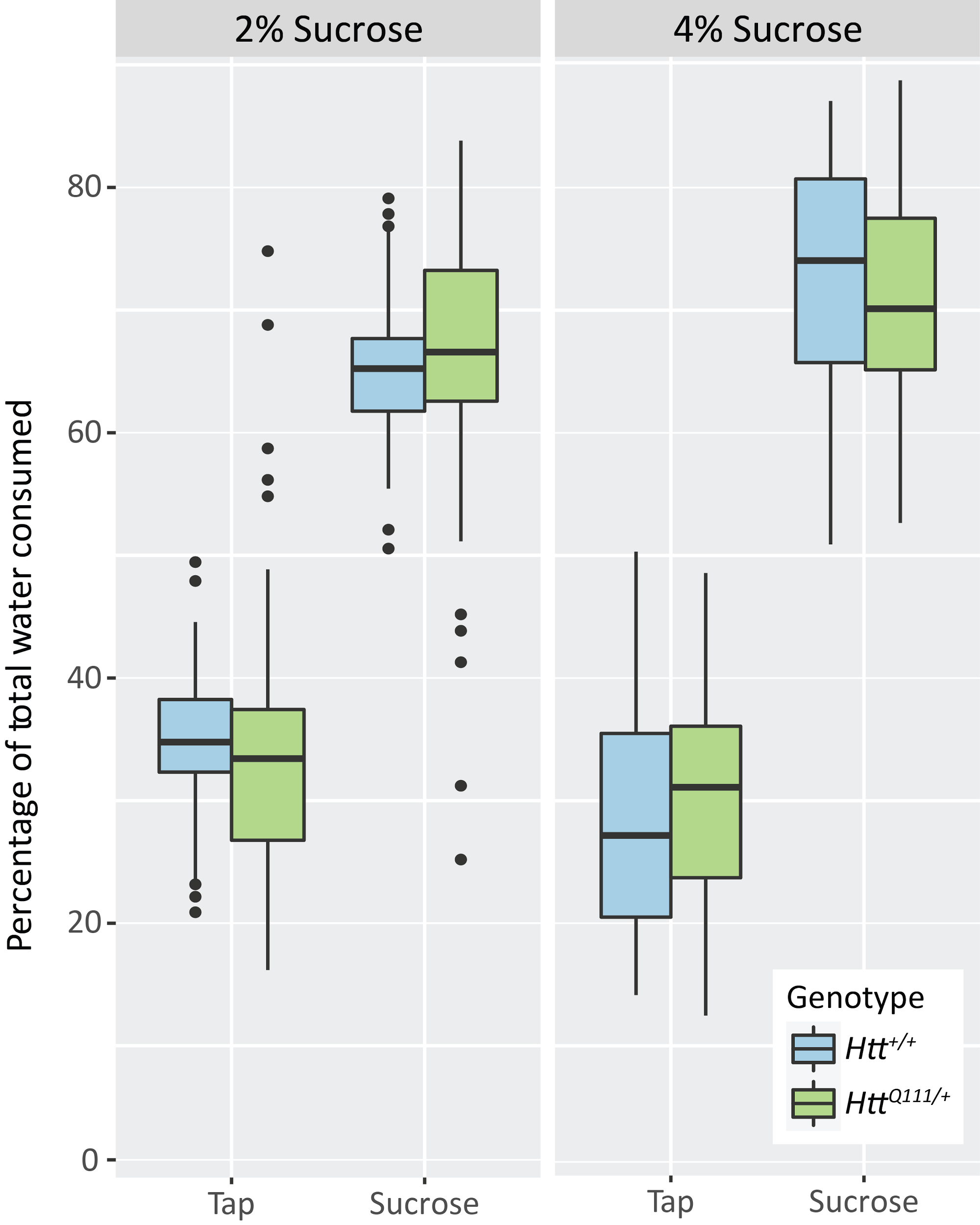


***Figure S1. Two-bottle sucrose preference test reveals no difference in sucrose preference between Htt^+/+^ and Htt^Q111/+^ mice.*** *Data are presented as boxplots.*

###

###

###
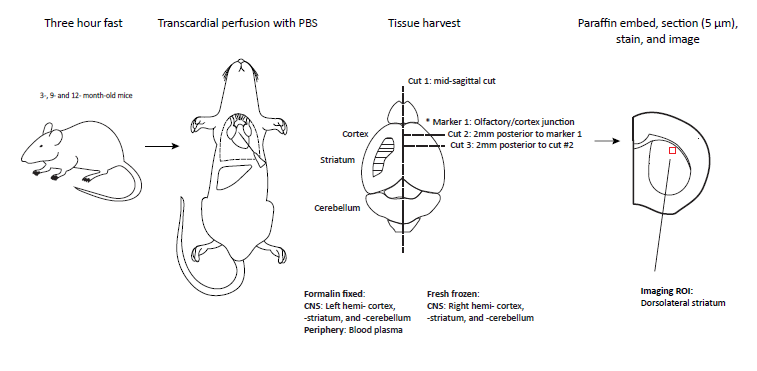


### ***Figure S2.*** *Tissue harvest workflow*
